# Preparation and Color Performance of White Ultra-High-Performance Concrete with Large Fraction of Quaternary Binders

**DOI:** 10.3390/ma15248895

**Published:** 2022-12-13

**Authors:** Rui Ma, Ziyang Tian, Wei Zhang, Lei Chen, Jinyu Zong, Yi Ding, Daosheng Sun

**Affiliations:** 1 Anhui Province Engineering Laboratory of Advanced Building Materials, Anhui Jianzhu University, Hefei 230022, China; 2 Anhui Sanjian Engineering Co., Ltd., Hefei 230001, China

**Keywords:** white ultra-high-performance concrete, quaternary binders, whiteness, mechanical strength, hydration products

## Abstract

White ultra-high-performance concrete (WUHPC) performed outstanding mechanical, durability, and aesthetical properties, which was preferred in infrastructure to avoid the secondary painting, decrease the maintenance, and prolong the service life. Supplementary cementitious materials (SCMs) were often used in WUHPC to reduce the environment impacts and material costs. In this study, limestone powder (LP), metakaolin (MK), and silica fume (SF) were used as SCMs to largely substitute white Portland cement (WPC) to prepare WUHPC, the effects of substituted ratio on flowability, strength, and whiteness were studied, and the hydration products were also analyzed by quantitative-XRD method and SEM. The whiteness was calculated in chromatic space CIELAB by measuring tristimulus values of L, a*, and b*, and the controlled factor on whiteness was also investigated. As the results, the WUHPC with compressive strength exceeded 150 MPa and whiteness over 90 was prepared with WPC substitution of 35~65%. The SF improved the flowability and strength about 10% due to its filling and ball effect, while the irregular particle sharp and non-uniform size distribution of MK caused the reversed development. The increased dosage of raw materials with higher L value, such as LP and MK, made the WUHPC whiter. The hydration products with varied SCMs ratio were in the same category by different content. It was supposed that CaCO_3_ and C-S-H gel in hydration products caused higher whiteness, while C_3_S, CaMg(CO_3_)_2_, and SiO_2_ were against the whiteness. The results proved that with a large fraction of SCMs, the WUHPC with high strength and good appearance were prepared, and the whiteness of WUHPC were both controlled by the raw materials and the content of hydration products.

## 1. Introduction

With the rapid urbanization and increasing demands for living, the requirements on concrete with durability and aesthetics performance have drawn more attention in recent decades [1,2,3]. For ordinary concrete buildings, a secondary surface painting is normally applied to keep a good appearance and prevent environmental corrosion [4,5]. However, the degradation, including aging, swelling, and spalling, mainly happened for painting pigment, which resulted in frequent maintenance and declined the service life of buildings [6,7]. White concrete is an energy-saving decorative construction material with an elegant look, is free from secondary painting, and has the highest reflection efficiency on light and heat [8,9]. White concrete serving as the out-wall of buildings could avoid the flaw of pigment and save the cost of maintenance and repair work. However, up to now, most research and applications of white concrete were focused on its artistical appearance as a non-bearing structure. To promote the application of white concrete in more bearing structures, the mechanical and the durability properties should have more emphasis to satisfy the increasing demands of buildings in further development.

White ultra-high-performance concrete (WUHPC) is a potential cementitious material which combines genes on extreme high strength and durability from UHPC and the aesthetical performance of white concrete [10,11,12]. Compared with normal white concrete, WUHPC can serve as a protective layer of normal concrete to improve the strength and corrosion resistance, repaired materials to reduce the maintenance cost, and thin bearing-structure due to its ultra-high strength [13]. Until recently, the research on WUHPC has been limited. Pyo et al. [14] prepared white UHPC using quartz-based mine tailings, and the flowability and the strength was slightly decreased compared to that with silica sand. Mo Alkaysi and Sherif El-Tawil [15] tried different raw materials to promote the low cost and studied the variations on compressive and tensile behavior. The results proved that white cement yielded the high compressive strengths, but also increased the material cost. Kim [13] constructed Façade using thin prefabricated white UHPC and studied its impact properties. Wenchen et al. [16] showed the creep of aged concrete under extremely high sustained load and investigated the behavior of transverse reinforcement on the reinforced concrete creep during the loading and sustained load process. The data, such as a higher transverse reinforcement ratio, can increase axial load capacity and reduce the concrete, especially in the early loading stage, which was of great help for this research. However, the dominant factors on color express of WUHPC are still unclear now.

White Portland cement (WPC) was essential in WUHPC to supply enough whiteness and strength [17,18,19]. However, the higher calcination temperature and longer calcination time during WPC manufactured process increased the material cost and the environment impact with CO_2_ emission [20]. Supplementary cementitious materials (SCMs) were commonly used to decrease the WPC dosage to solve the above problems [21]. Sharaky et al. [22] demonstrated that with more active ingredients in metakaolin (MK), like SiO_2_ and Al_2_O_3_, the strength of high-strength concrete first increased then decreased, and reached the maximum with 15% WPC substituted. Ferraro et al. [23] proved that when using rice hush ash to replaced WPC less than 15%, the chloride ion diffusion coefficient was reduced by refined pore structure, and the strength was slightly lost. Liu et al. [24] used silica fume (SF) to equivalent replace cement, and found that when SF content was 10%, the workability of the concrete was improved, while the strength was reduced. This change was reversed with 20–30% SF. However, considering both the mechanical strength and appearance, the effect of SCMs on both aesthetic properties and particle packing density should be carefully determined.

In this study, limestone powder (LP), metakaolin (MK), and silica fume (SF) were selected as SCMs, and WUHPC with quaternary binders was prepared. The effects of materials mix proportion on flowability, mechanical properties and aesthetic properties were investigated, and the hydration products were also analyzed to reveal the mechanism on appearance.

## 2. Materials and Methods

### 2.1. Raw Materials

**Figure 1:**
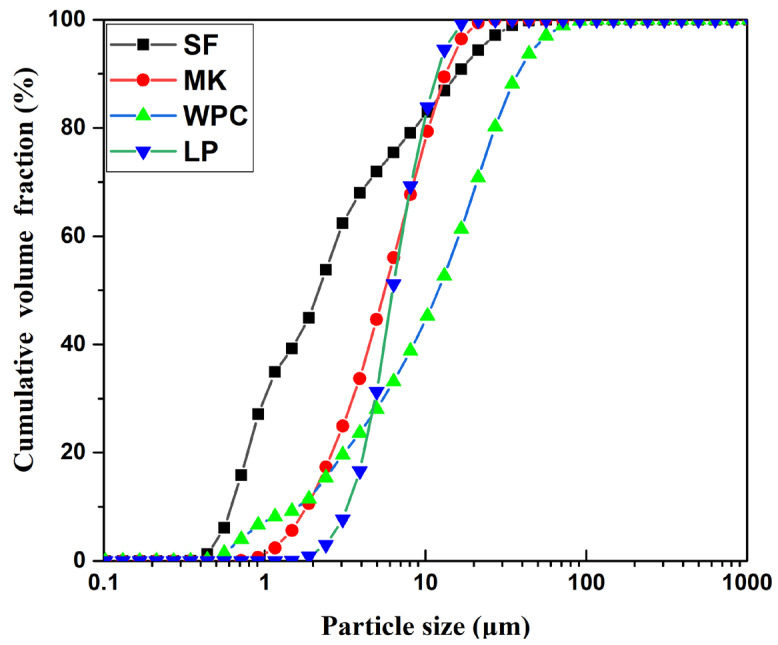
Particle size accumulates cures for binders.

To decrease the cost and the CO_2_ emission, White Portland cement (P·W 52.5, WPC), limestone powder (LP), metakaolin (MK), and silica fume (SF) were selected as binder materials to prepare WUHPC. The chemical composition of binders is listed in Table 1. The particle size distribution of binders is shown in Figure 1. River sand with a fineness modulus of 2.6 was used as fine aggregate, and 13 mm length steel fiber with a length-to-diameter ratio of 60 was selected as the reinforced materials. The mechanical properties of steel fiber are summed up in Table 2. Polycarboxylic acid superplasticizer with a water-reducing rate of about 30% was involved to improve the flowability of WUHPC. For comparation, normal UHPC were also prepared as a control group from our previous work [25].

To investigate the effect of material proportions on the properties of WUHPC, water to binder ratio (W/B), LP%, MK%, and SF% were selected as variants in an orthogonal experiment. The change levels for each variant are shown in Table 3. The mixing proportion is listed in Table 4.

### 2.2. Mixed Process and Curing Regime

During the mixing procedure, the powders, including binders, sand, and superplasticizer, were first dry-mixed for approximately 1.5 min, then water was dropped into the mixture with the continuously stirring about 5–7 min until the uniform mixture was formed. Fiber was added last and was stirred for another 2 min for good distribution.

After the matrix was ready, it was casted and curried under standard condition (20 °C ± 5 °C, RH > 95%) for 1.5 days, then demolded and steam-cured at 85 °C for 3 days.

### 2.3. Test Method

**Figure 2:**
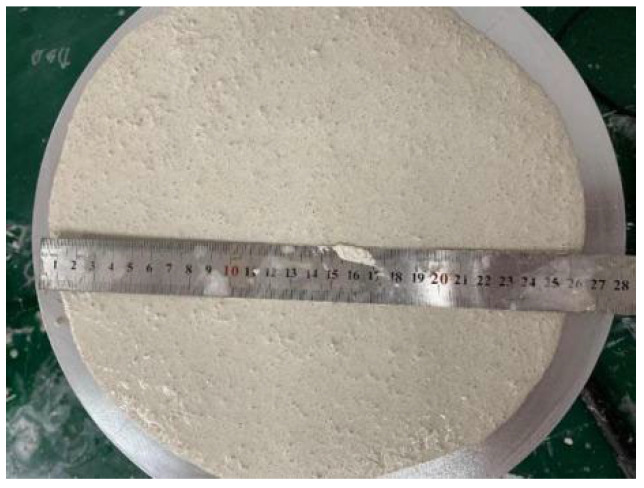
Test setup for flowability.

The flowability was tested followed by Chinese standard “Test method for fluidity of cement mortar” (GB/T2419-2005), the fresh matrix was cast into a cone mold and placed on the platform of a jolting table, then the mold was removed vertically and jolted for 25 times. Two dimeters at vertical direction after jolted were measured as the flowability. The test method is shown in Figure 2.

**Figure 3:**
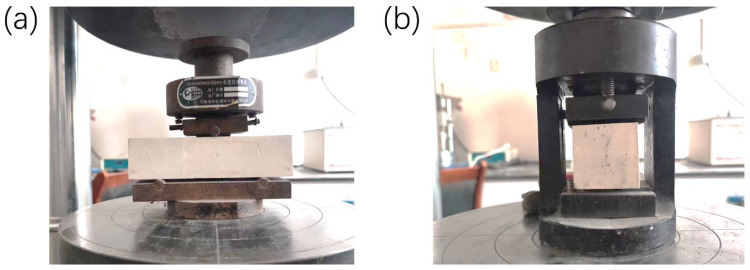
Experimental test setup for flexural strength (**a**) and compressive strength (**b**).

The strength of WUHPC was tested according to Chinese standard “Method of testing cements—Determination of strength” (GB/T 17671-1999), a three-point bending test was taken for the prism specimens with the size of 40 × 40 × 160 mm to determine the flexural strength, and an axial compress load was applied at the end of each broken specimen for compressive strength after flexural strength tests. The strength test setup is shown in Figure 3.

**Figure 4:**
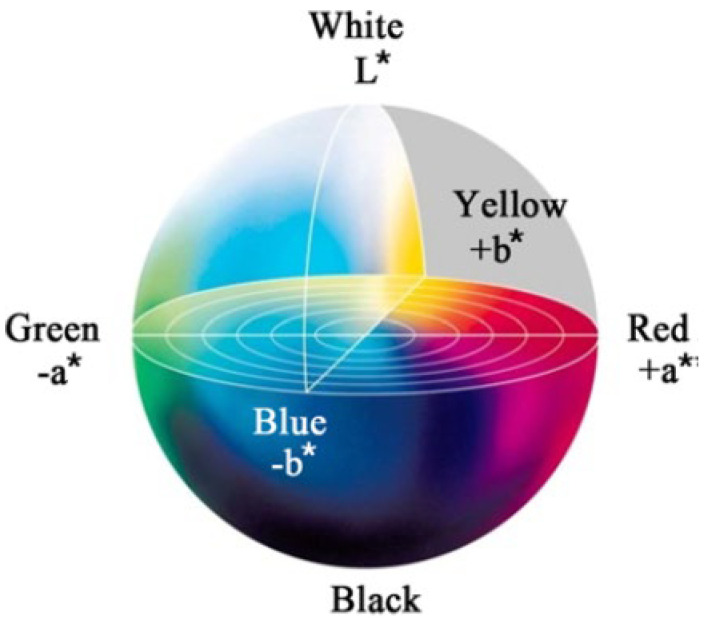
Lab color coordinates [26].

The color of WUHPC was evaluated by CIELAB space with tristimulus value *L*, a* and b* [18,26,27], as shown in Figure 4. In CIELAB, *L* defines the brightness from completely opaque (0) to completely transparent (100), a* is a measure of redness (−a* greenness), and b* is a measure of yellowness (−b* blueness). During the test, each value was measured by a colorimeter at 3 points on the surface of specimens for an average. The whiteness was calculated by Equation (1) as below:

HW = 100 − [(100 − *L*)^2^ + a*^2^ + b*^2^]^1/2^ (1)

where HW referred to Hunter Whiteness.

The hydration products were analyzed by the quantitative X-Ray diffraction (XRD) method, and the morphology of WUHPC was observed by scanning electron microscopy (SEM). To avoid the interference from SiO_2_ and steel, paste specimens with no sand and fiber were prepared under the same curried regime. Before the quantitative XRD test, the paste specimens were firstly ground to be smaller than 80 μm, then 10 wt% of Al_2_O_3_, used as the internal standard material, was added, and was fully ground to be well-dispersed. During the test, the sample was scanned from 5–75° (2θ) with the step of 10°/min. The phase content in the test was calculated with Equation (2):

(2) $$X_{i}=[X_{s}K_{s}^{i}(1-X_{s})]\cdot (I_{i}/I_{s})$$

where *X_i_* and *X_s_* are the content of material *i* and internal standard material *s* (wt%), $K_{s}^{i}$ is the *K* value of material *i* to *s*, and *I_i_* and *I_s_* are the diffraction intensity of *i* and *s*, respectively.

## 3. Results and Discussion

### 3.1. Fresh Properties for WUHPC

**Figure 5:**
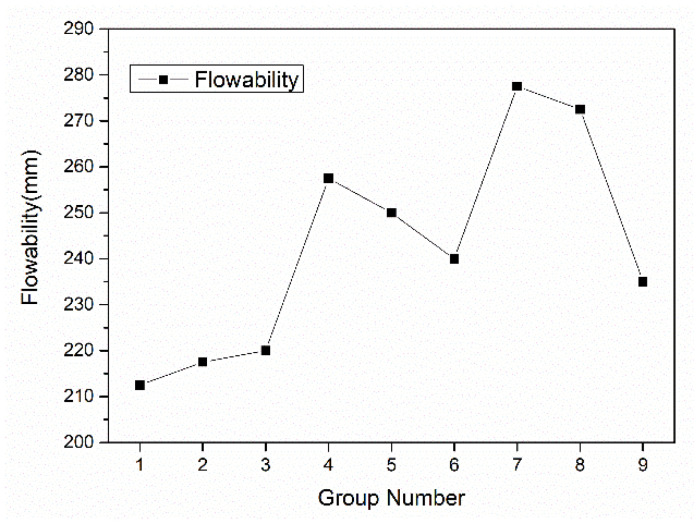
Flowability of WUHPC with varied factors.

The flowability of fresh mortar with different W/B and SCMs ratio is shown in Figure 5. The average value of fluidity corresponded to W/B and each binder ratio is summarized in Table 5. It was seen that flowability was greatly influenced by W/B. Once W/B increased 0.15 to 0.17, the fluidity was also increased from 216.67 mm to 261.67 mm, about 21%. Still, flowability also improved with more SF. It rose from 232.5 mm to 250 mm once SF was added from 5% to 15%. However, with the increase of LP, flowability first slightly decreased, then increased. The addition of MK caused the continuous decrease of flowability.

**Figure 6:**
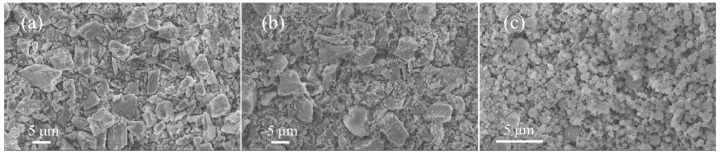
SEM images of mineral admixtures: (**a**) LP, (**b**) MK, and (**c**) SF.

It is easily understood that higher W/B improved flowability due to the flow effect of more water. For binders, the flowability was adjusted by their particle morphology and packing density. As shown in Figure 1, the size of SF ranged from 0.4 μm to 50 μm, which was much smaller than other binders, and the particles that were less than 10 μm were in dominated fractions. It could occupy the voids generated from particles packing and increased the compaction of matrix and leave more water to lubricant so that the flowability was increased with more SF dosage. On the other hand, the morphology of mineral admixture is present in Figure 6. It can be seen that the particles of LP and MK were irregularly shaped, while SF particles were all small balls. The grain of MK was even less, because the size of particles was either too large or too small, and it consumed more water to wet particle surface during mixing. The flowability was decreased with the addition of MK. The ball effect of SF was also beneficial for flowability.

### 3.2. Mechanical Properties

**Figure 7:**
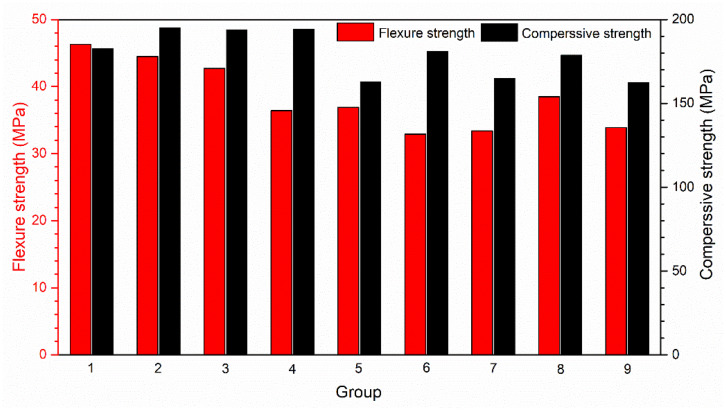
Flexural and compressive strength of WUHPC.

Compressive and flexural strength of WUHPC with different binder contents are shown in Figure 7. It can be seen that the strength of WUHPC was magnificent, even with a high-volume fraction of substitutions. The compressive strength all exceeded 150 MPa after 3 days of steam curing, even reached 195.1 MPa at maximum with a WPC substitution ratio of 50%. The detailed data are also listed in Table 6. It was found that the flexural and compressive strength were both decreased with the increase of W/B. Additionally, the raised ratio of SF from 5% to 15% enhanced the compressive strength by about 11.5%. Otherwise, the change of substituted ratio of binders caused the strength to insignificantly waver by less than 5%.

Higher W/B induced more water in WUHPC. After the water was consumed and evaporated, the occupied space changed into voids and cracks in the matrix, which was harmful for strength development. The compressive strength was decreased with higher W/B. In addition, the reactivity of mineral admixture was generally lower than cement. The substitution diluted the concentration of WPC, leading to a decrease in strength. However, SF with small particles filled the blanks to compact the matrix, and the pozzolanic reaction generated more C-S-H gel, which improved the microstructure to enhance the strength. Although MK was rich in SiO_2_, the activity was much lower than SF because of the high crystallinity. In addition, the primary phase in LP was CaCO_3_, which reacted in cement as in Equations (3) and (4). The hydration products were crystal with no cementitious properties.

C_3_A + 0.5 CaCO_3_ + 0.5Ca(OH)_2_ + 11.5H_2_O → C_4_AC_0.5_H_12_ (3)

CaMg(CO_3_)_2_ + 2MOH → CaCO_3_ + Mg(OH)_2_ + M_2_CO_3_ (4)

where M denotes K^+^, Na^+^, or Li^+^.

### 3.3. The Aesthetics Performance of WUHPC

**Figure 8:**
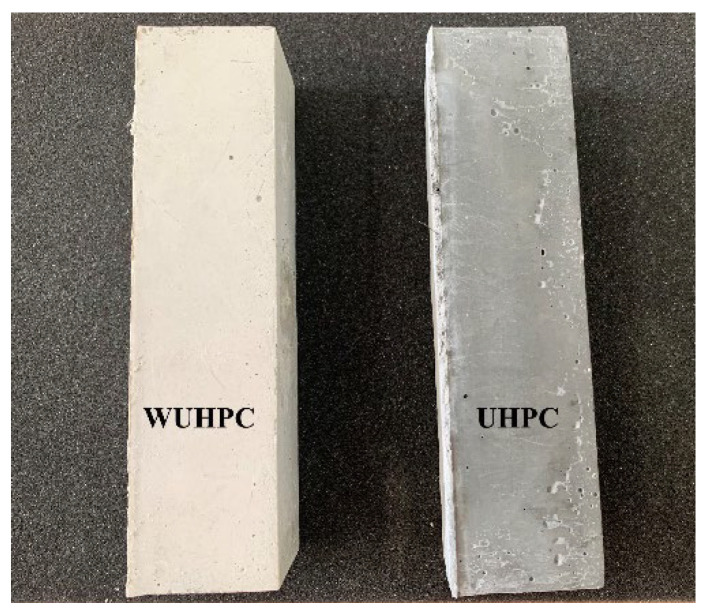
Appearance of WUHPC and normal UHPC.

**Figure 9:**
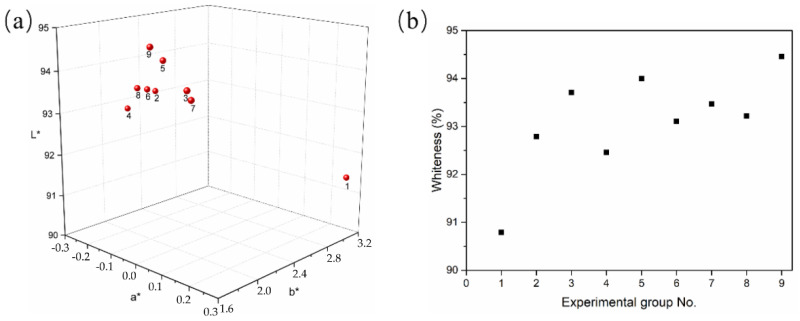
The (**a**) 3D dispersion of tristimulus values and (**b**) whiteness of WUHPC.

The appearance of WUHPC and normal UHPC is compared in Figure 8, and the aesthetic of WUHPC with a white and smooth surface was much better than normal UHPC. To understand the effect of binders on the whiteness of WUHPC, the test results of whiteness are summarized in Table 7 and Figure 9.

**Figure 10:**
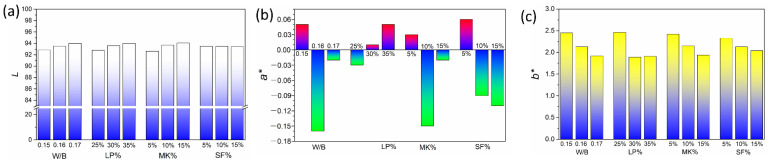
The effect of W/B and minerals addition on (**a**) *L*, (**b**) a*, and (**c**) b*.

From the results, WUHPC samples were all good-looking with the whiteness ranging from 90.79 to 94.46, which was approximately double normal UHPC (control). Still, the whiteness vibrated with the difference according to W/B and minerals contents. To investigate the regulation detail of whiteness, the effect of admixture binder proportion on *L*, a*, and b* in CIELAB system was studied as shown in Figure 10.

In Table 7, it can be seen that the whiteness of WUHPC was mostly determined by *L* value, and the higher *L* resulted to more whiteness. As shown in Figure 10a, the *L* value was continuously increased with the increase of W/B, LP%, and MK%, but not affected by SF%. In addition, the b* declined with the increase of W/B and minerals addition (Figure 10c), which meant that the color was less yellow and also contributed to whiteness. However, the a* was changed irregularly with the W/B and minerals dosage variation.

Since the W/B in WUHPC was much lower, raw materials were not fully hydrated. The color was affected by residue materials. In Table 7, the *L* value of binders was WPC < SF < MK < LP. With the increase of the WPC substituted ratio, the *L* value of WUHPC was increased, and this effect was more significant when higher fraction of mineral admixture with large L existed. Additionally, with the higher W/B, the hydration degree of WUHPC was promoted, and more hydration products were formed. However, compared with the sample 3, 5, and 9, it seems that with a higher hydration degree, even if more admixture with larger L was added, the whiteness of WUHPC was smaller. The results proposed that the hydration products were also affected by the whiteness of WUHPC.

To further investigate the effect of hydration products on whiteness, we examined the paste samples by XRD.

### 3.4. The Hydration Products in WUHPC

**Figure 11:**
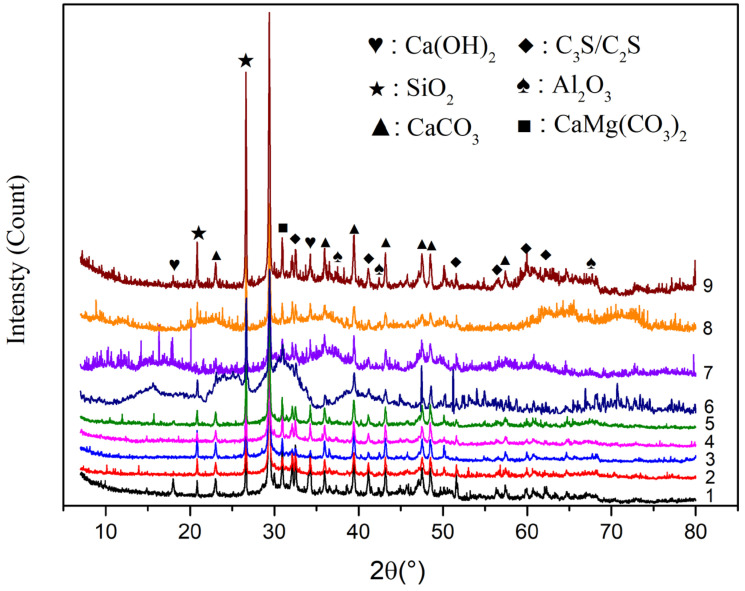
XRD pattern for typical WUHPC sample.

The XRD patterns for WUHPC pastes are shown in Figure 11. The main diffraction peaks are marked in the picture. They can be divided into two categories. One was from raw materials, for instance, CaCO_3_ from limestone, C_3_S/C_2_S contained in WPC, and SiO_2_ from MK. The other was hydration products, mostly Ca(OH)_2_ and some calcium aluminate hydrates and calcium sulfoaluminate hydrates, which were not marked with low intensity and complex diffraction peaks. The diffraction of Al_2_O_3_ was the internal standard substance.

It was found that the category of diffraction peaks in different samples was the same, which means that the varied ratio of SCMs did not change the hydration reaction to generate additional products. However, the intensity of diffraction peaks was different in samples, implied that the concentration of products may be different. The quantitative content of crystal phases, calculated according to calibration Al_2_O_3_, is listed in Table 8.

The content of Ca(OH)_2_ increased with the higher W/B, proving that the hydration degree was promoted with more water. Additionally, with the further hydration degree, more alkali formed to promote the reaction. The CaCO_3_ and CaMg(CO_3_)_2_ content in group 7 was lower than that in group 3 and 5.

Gel products like C-S-H were generated during cement hydration but were not detected in XRD patterns. This can be calculated indirectly by the sum mass of specimens. In Table 6, the sum of weight fractions of primary crystals was not 90% (calibration Al_2_O_3_ was 10 wt%), and the residue was supposedly the gel products. With the W/B raised, the gel content was increased, which meant that the hydration process was promoted.

Corresponding to whiteness, it was supposed that CaCO_3,_ CaMg(CO_3_)_2_, and C-S-H gel contributed towards higher whiteness, while the existence of C_3_S and SiO_2_ was against the whiteness.

Iker Marcaida [28] investigated the chemical composite of pigment with different color, and found that dolomite CaMg(CO_3_)_2_ determined the white color. Enpei Ma [29] used SiO_2_ to encapsulate paraffin to fabricate colored microcapsules.

**Figure 12:**
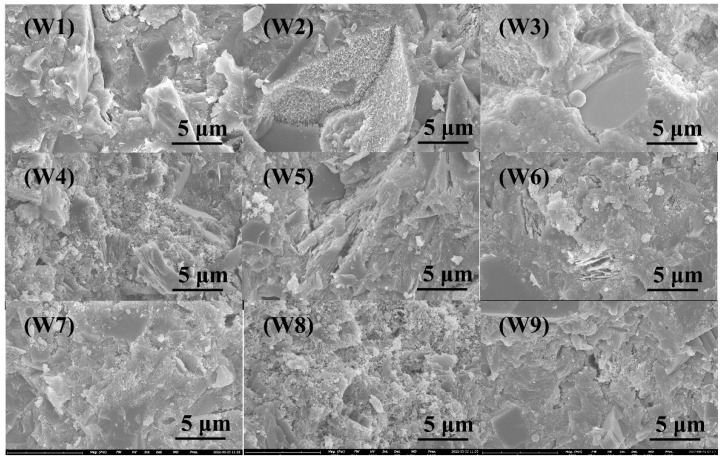
SEM images for WUHPC of sample groups W1–W9.

The SEM images of different WUHPC samples are presented in Figure 12. From images, the matrix of WUHPC became loose and more clusters of products formed with the increase of W/B. Under the same W/B, the matrix was denser with a higher content of LP. Still, with the addition of MK, more sheet form products were found in WUHPC, which induced more cracks and defects.

## 4. Conclusions

In this experiment, limestone powder (LP), metakaolin (MK), and silica fume (SF) were used as SCMs to substitute white cement to prepare white ultra-high-performance concrete (WUHPC), and the WUHPC with good mechanical strength and appearance was successfully fabricated with a high fraction of SCMs. The effect of substituted materials ratio on flowability, strength, and color expression were investigated, and some conclusions were drawn below:

(1) The compressive strength of WUHPC was all over 150 MPa, with a fraction of SCMs ranging from 35% to 65%, reaching 195.1 MPa at maximum with 50% cement substitution. The whiteness of WUHPC exceeded 90, which was about two times higher than that of normal UHPC.
(2) The increased ratio of SF improved the flowability and compressive strength of WUHPC due to the filling and ball effect by small and rounded particles, while the irregular sharp and non-uniform particle size distribution of MK decreased the flowability, but insignificantly affected the strength.
(3) The aesthetics properties of WUHPC were evaluated by CIELAB space. Because of low hydration degree, the color of raw material almost determined the color of WUHPC, higher L value of raw materials lead to higher whiteness, and the increased ratio of LP and MK was good for whiteness increasing. The higher hydration degree caused by larger W/B also increased the whiteness of WUHPC.
(4) Based on quantitative-XRD analysis, the mineral admixture did not change the species of hydration products but varied the content. The existence of CaCO_3_, CaMg(CO_3_)_2_, and C-S-H gel was preferred for higher whiteness, while a higher content of C_3_S and SiO_2_ resulted in a relative lower whiteness.

## Figures and Tables

**Table 1:** Chemical composition (wt%) of experimental materials.

Material	CaO	SiO_2_	Al_2_O_3_	Fe_2_O_3_	MgO	Na_2_O	K_2_O	P_2_O_5_	SO_3_	L.O.I
WPC	67.09	18.09	2.25	0.27	4.49	0.30	0.68	0.01	4.33	2.48
SF	0.30	95.87	0.50	0.42	0.34	0.13	0.04	1.25	1.01	0.13
MK	0.48	95.59	0.90	0.06	0.38	0.05	0.12	0.01	2.35	0.06
LP	92.26	0.20	0.03	0.03	0.27	--	--	0.03	0.02	7.16

**Table 2:** Specification of used fibers.

Type	Length/mm	Aspect Radio	Tensile Strength/MPa	Picture
Straight steel fiber	13	60	2850	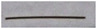
Hooked steel fiber	13	60	2850	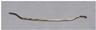

**Table 3:** The variate design for orthogonal experiment.

No.	W/B	LP %	MK %	SF %
1	0.15	25%	5%	5%
2	0.15	30%	10%	10%
3	0.15	35%	15%	15%
4	0.16	30%	5%	15%
5	0.16	35%	10%	5%
6	0.16	25%	15%	10%
7	0.17	35%	5%	10%
8	0.17	25%	10%	15%
9	0.17	30%	15%	5%

**Table 4:** Mixed proportion design (kg/m^3^).

No.	WPC	SF	MK	LP	Total Binders	Super-Plasticizer	Water	Steel Fibre		Sand
								Straight	Hook	
W1	701	54	54	270	1079	15	167	136	68	1079
W2	540	108	108	323	1079	15	164	136	68	1079
W3	377	162	162	378	1079	15	162	136	68	1079
W4	540	162	54	323	1079	15	172	136	68	1079
W5	539	54	108	378	1079	15	174	136	68	1079
W6	539	108	162	270	1079	15	173	136	68	1079
W7	539	108	54	378	1079	15	181	136	68	1079
W8	539	162	108	270	1079	15	180	136	68	1079
W9	539	54	162	323	1078	15	182	136	68	1079

**Table 5:** Range analysis of fluidity (mm).

Change Level	W/B	LP %	MK %	SF %
K_I_	216.67	241.67	249.17	232.50
K_II_	249.17	236.67	246.67	245.00
K_III_	261.67	249.17	231.67	250.00

**Table 6:** Range analysis of flexural (F) and compressive (C) strength (MPa).

Change Level	W/B		LP %		MK %		SF %	
	F	C	F	C	F	C	F	C
K_I_	44.5	190.6	39.2	180.9	36.4	180.7	39.0	169.4
K_II_	35.4	179.5	38.3	184.0	40.0	178.9	36.9	180.4
K_III_	35.3	168.8	37.7	173.9	36.5	179.1	39.2	189.0

**Table 7:** The CIELAB index and Hunter whiteness of raw materials and WUHPC.

Materials		*L*	a*	b*	*HW*
Raw materials	WPC	91.24	0.30	3.11	90.70
	SF	94.51	0.34	0.12	94.50
	MK	97.22	0.57	1.29	96.88
	LP	98.14	0.53	0.23	98.05
Matrix	Control	54.83	−0.92	5.03	47.57
	W1	91.35	0.26	3.16	90.79
	W2	93.24	−0.22	2.37	92.79
	W3	93.98	0.11	1.83	93.71
	W4	92.80	−0.28	2.22	92.46
	W5	94.35	−0.05	2.02	94.00
	W6	93.46	−0.17	2.16	93.11
	W7	93.75	0.11	1.87	93.47
	W8	93.54	−0.17	2.06	93.22
	W9	94.77	−0.02	1.82	94.46

**Table 8:** Weight fraction of crystal phases according to XRD analysis (wt%).

Group	C_3_S	CaCO_3_	SiO_2_	Ca(OH)_2_	CaMg(CO_3_)_2_	Total
W1	17.3	17.0	7.5	4.9	10.2	57.0
W2	17.2	20.4	12.1	3.1	8.2	60.9
W3	13.0	27.1	19.0	3.5	8.8	71.4
W4	15.2	20.9	8.4	3.4	10.2	58.1
W5	14.8	23.4	11.9	3.6	9.3	62.9
W6	12.9	8.4	8.7	3.1	9.8	42.9
W7	12.9	11.0	4.5	4.2	7.8	40.5
W8	11.6	8.5	6.4	3.4	6.6	36.5
W9	12.5	15.3	12.2	3.6	8.2	51.7
